# The Different Phases of Electric Treatment

**Published:** 1899-12

**Authors:** J. McFadden Gaston

**Affiliations:** Atlanta, Ga.


### THE DIFFERENT PHASES OF ELECTRIC TREATMENT.*

By J. McFADDEN GASTON, M.D.,

ATLANTA, GA.

A thesis on Electric Therapeutics, in the Portuguese language formed the basis of my application twenty-five years ago for recognition by the Imperial Academy of Medicine, Rio de Janiero, Brazil.

I defended successfully the points presented in that paper on the electric treatment of diseases of various kinds before the Medical Faculty, and incidentally referred to the electric current as a fluid. One of the Professors raised an issue upon my use of the term fluid in this connection, stating that there was no similarity to water or other fluids. In my rejoinder to his criticism I remarked interrogatively: "If it is not a fluid, what is it?" And the Professor promptly said he was satisfied, and this closed my examination in his branch.

This question remains unanswered after the lapse of a quarter of a century, and while, like faith, electricity is the evidence of things not seen, it shows its fruits in works of the most potential order. All the effects of electricity depend upon this: the separation into positive and negative currents, terminating in corresponding electrodes. The development of a great force, which may be utilized either in the cure of diseases or industrial operations, has grown out of the mechanical appliances resorted to by experts in electrical works.

Preliminary to any details of the application of electricity as a therapeutic agent, it is proper to notice the various sources of electricity. Commencing with the experiments of Franklin with the kite and a wire, by which it was demonstrated that electricity passes from the clouds to the earth, practical observations upon this means of manifesting electric phenomena have been made which go to prove that all bodies are endowed with what may be styled

*Presented to the meeting of the Southern Surgical and Gynecological Association at New Orleans, December 6th, 1899.

latent electricity. This holds in regard to inorganic as well as organic bodies, and it only requires the resort to a certain process to develop manifestations of electricity. The Leyden jar is the most simple contrivance for exhibiting the local accumulation of the two elements of static electricity, which are kept apart by the non-conductor of glass, until a jointed metallic conductor is brought in contact with the inner and outer surface of the glass jar, which has a coating of tinfoil covering the lower portion of the jar. Static electricity is generated and collected by processes with which all who have been in a chemical laboratory are familiar, and needs no elucidation.

The other modes of generating electricity are recognized generally by the names of those who have invented apparatus suited to the different modes of manifestation, such as Volta, Faraday, and Galvani. Independent of these exhibitions of electric force the dynamic current is derived from a permanent electro-magnet, in which the current is of an alternating character. This form of current possesses great electro-motor force, but is practically the same as the Faradaic current, and not so well adapted to the treatment of diseases.

I must take it for granted that the first principles of electric treatment are understood, as it is not within the scope of this paper to go into any detailed account of the apparatus used in connection with the various manifestations of the electric current.

The terms employed to qualify the action on the tissues are electrolysis and cataphoresis, the latter being the entrance of medicines from the positive electrode of the battery.

The continuous current is employed in applying electricity for the modification of the abnormal structure, and the negative pole is brought in contact with the tissue of the part or organ to be acted on in the process of simple electrolysis. This is accomplished in most cases by using a needle as negative electrode, inserted into the affected tissues. It is found that with the transmission of the Galvanic current, the tissue into which this enters becomes disintegrated and that small bubbles of gas escape around the needle, so that it gradually becomes loosened, thus breaking down the indurated structure.

When it is desired to convey any medicinal influence through a part that is diseased, a solution of the medicine is placed upon the pole of the positive electrode, and with the action of the electric current the medication is transmitted to the intervening tissues so as to make its appropriate impression.

The interrupted induced current from a Faradic battery is usually applied in cases of impaired nerve power and paralysis or paresis, in which order of debility, not dependent upon any radical organic change in the nerve centers, we may expect benefit. In certain forms of neuralgia and in rheumatism the inductive process may be relied on for relief in connection with medication internally and massage over the region involved.

The favorable action of a horse-shoe magnetic application in some neurotic conditions is due to the recognized fact that a magnet is a magnet by virtue of currents of electricity circulating around the central mass of iron.

There are many variations of so-called electric belts and appliances which do their work upon the imagination without effecting any material change worthy of note.

The adaptation of the various forms of electricity to different diseases of a medical and surgical character has encountered opposition by gynecologists on account of the alleged adhesions of the structures involved. It is claimed that in the event of a failure to arrest the disorder and that a surgical measure should be found necessary, complications are met with from the agglutination of the tissues. That benefit is derived in some cases of uterine fibroids by the resort to electricity is generally admitted, but it is claimed by those opposed to its use that more harm results from its application than benefit. It should be noted that those having most faith in the electric treatment are applying it to a great variety of cases and thus speak from experience of its good effect, whereas those opposed to it, refuse to put it to the test of actual use in their practice. Practical application and not theory should settle this matter.

The special favorable results of the use of electricity in extra-uterine pregnancy obtained by Thomas and by other gynecologists, prior to the rupture of Fallopian tube or sac elsewhere of the adnexa, should be limited to the death of the fetus. It is not understood to extend to the absorption of the components of the mass, but simply to arrest of growth by death of the fetus. It is evident that changes take place subsequently which would render an operation less dangerous in some cases, but on the other hand decomposition of the dead fetus may lead to the collection of pus which assumes the nature of a diffuse abscess in the abdominal cavity accompanied with contamination of the whole system, and in the end terminating fatally. The writer has met with such a case that was verified by a post-mortem examination and accompanied by the most offensive odor which has been encountered in any other collection of a purulent nature.

The resort to laparotomy in ectopic gestation has been attended with good results in so many instances at various stages of pregnancy as to authorize section in suitable cases, whether the fetus be dead or alive; and it is not precluded by a previous use of electricity for the arrest of fetal development.

The class of cases in which electrolysis and cataphoresis have been employed are generally of a benign character; but even malignant growths have been removed without resorting to the knife or escarotics of any kind. The use of a destructive process with an electric cautery has also proved effective in inoperable tumors of a malignant nature and has advantages over those of a chemical order, such as caustic potash and arsenical paste.

Betton Massey, of Philadelphia, has the credit of introducing the electric cautery as a destructive agent in the treatment of malignant growths, and his results have proved satisfactory.

It is not my purpose in this paper to give any extended notice of the results obtained by electric treatment, but to refer briefly to the class of cases to which it has been applied with benefit.

The simpest application of the disintegrating process effected by the continuous current through a needle constituting the negative electrode, is in the removal of hairs from the face, which, when done with proper precautions, do not return.

With a suitable electrode on the negative pole strictures of the urethra, the rectum, and esophagus are relieved greatly, if not entirely cured, by the continuous current of galvanism.

Enlargement and induration of the prostate gland are treated successfully by the insulated negative electrode, and without any considerable discomfort.

Nevus and cirsoid aneurism in which the blood-vessels are unduly developed are found to yield under the use of the electric current connected with the needle introduced around the border of the growth of vascular tissue so as to cut off the circulation.

In the treatment of vascular tumors of all kinds by electricity, the favorable result depends upon the local coagulation of blood with the formation of thrombi in the affected mass.

Sacculated and other forms of large aneurism have yielded to the punctures with needles on each side and the passage of electrity through the sac and contained mass of blood.

Epithelioma, sarcoma, and lupus are undergoing the crucial test of clinical observation upon the results of the electrolytic process in connection with special application of active medication through the positive pole.

R. N. Fraser has recently reported his case of recurring malignant disease of the scrotum by Gaston's method of cataphoresis and the internal administration of Donovan's solution. Seven recurrences were promptly treated by the knife, and followed by this method until a cure with no recurrence for fifteen months resulted. He considers that a trial should be given all cases of recurrent disease, and no operative measure withheld at the same time to aid and supplement the action of electricity.

But, above all, early treatment is necessary in tumors of a malignant type, and it is my candid opinion that many cases will yield to electricity systematically administered in the way that Betton Massey, Fraser and myself have indicated. It has been, however, my experience that people were referred to me when all the lymphatic glands were thoroughly poisoned and when the rapid disintegrating process precluded the idea of relief by electricity.

Even the more radical measures advised by Betton Massey meet with success when resorted to early in the treatment of malignant tumors.

His results with tumors on the breast coincide in the main with mine in cases of sarcoma and carcinoma, where the microscope left no doubt as to the character of the neoplasm.

In tumors of the breast a disposition to recurrence must be met by constant and repeated applications of electricity. Cases in which cervical endometritis yielded to a limited number of applications of electricity would convince me of the great efficacy of the local application of Lugol's solution of iodine and iodide of potassium to the cervix at the negative electrode.

Such cases are amenable to treatment with intrauterine electrodes and the Apostoli clay electrode on the abdomen, with the Apostoli bi-polar electrode, or with the Goelet intrauterine electrode and a small round zinc amalgam or copper electrode externally.

A state of chronic invalidism with loss of flesh, constant pain, and the most marked despondency, may give way to perfect health, an increase of adipose tissue, relief of pain, and buoyant spirits. But regular and systematic treatment, attention to the condition of the bowels, and to the habits of the patient should be regarded.

These cases of cervical endometritis may be mistaken for leucorrhea, and even gonorrhea, and it is necessary to examine digitally or by speculum, for erosion, cord-like condition and protrusion of the cervix.

Another class of cases presenting a phase of electric treatment consists of ulcerated patches due to epithelioma or varicose veins or any lack of proper vascularity of the tissues. These eroded and suppurating sores differ from the ordinary effects of inflammation in the peculiar indolent character of the skin. Very many of these show no tendency to spread, but remain for months and years almost stationary. The application of the electric current to the skin supplies a stimulus to the vaso-motor nerves, and a new growth of cells may result.

In these cases Donovan's solution may be used by cataphoresis and is an excellent antiseptic and germicide. At the same time blisters are the result of a strong current with the electrodes within a few inches of one another. These blisters at times give some concern to the patient, but are uniformly satisfactory, forming simply a dense scab resembling a button, even when quite extensive.

Many cases of atrophy of the muscles are due to deficient innervation and only require an active agent like Lugol's solution to respond to treatment thereby.

Techsner has reported such cases to be benefited by certain gymnastic exercises, but in the case of a man earning his living by the exercise of these muscles a sufficient use of them may be already present, and only a comparatively penetrating agent, such as molecular decomposition is, may arrest further atrophy.

Hemorrhage from the nose is a frequent symptom of malignant tumors of the nostril and yields readily to the interpolar action of electricity. Granulation may progress favorably when aided by a decomposition of cells already thrown off in the granulating progress of repair.

But it is in inoperable tumors and remote and obscure masses of tissue, such as stricture of the esophagus, rectum, or urethra, aneurism of the aorta and angioma of the mediastinum that the current of electricity holds a place by itself.

In many vascular masses and surfaces the positive needle may be necessary to arrest hemorrhage, but as a general rule, the needle should be used on the negative pole, while the sponge electrode upon which Donovan's solution is applied, may be the positive.

The cases of goitre which are most directly affected by cataphoresis are the parenchymatous enlargements of the thyroid gland. The so-called cystic variety is not so readily disintegrated and may be better treated by the needle if the electrical current is used.

Hunter McGuire, of Richmond, reported some very interesting results obtained with tincture of iodine, applied on cotton at the positive electrode, in doses of from ten to fifteen drops.

The first of these cases was in an old person, and the applications were made daily for several weeks. After a certain time the thyroid had decreased to one-fifth of its former size. The gland then seemed to remain stationary and to resist further treatment. But two other cases were entirely cured by McGuire and so reported in the *Virginia Medical Monthly* in August, 1891.

It was partly due to these results that I undertook to treat all malignant growths by cataphoresis.

The cases of goitre coming under my personal observation have been treated after the manner of McGuire, using Lugol's solution by preference. In cases where Lugol's solution was applied to the negative pole a general erythema occurred at this electrode and the skin afterwards was excoriated and denuded; at times, even blistered.

For gentle treatment I would advise the use of two milliamperes and of a small dosage of iodine and iodide of potassium. The latter drug is a test for the positive pole, where a brown color will occur if a saturated solution is traversed by a direct current of 110 volts.

In the management of the direct current a few lamps may be used in circuit so as to decrease the amperage. Twenty 16-candle power lamps in series will change an ordinary incandescent lamp light so as to be scarcely visible, and the current of electricity, which is generally one-half ampere, to one-fortieth of one ampere. This is equivalent to $\frac{1}{4}$ of 100 milliamperes, or 25 milliamperes.

This is a very useful average to establish in beginning the treatment of fibroids, or of sarcoma by cataphoresis. But after a week of such applications, eleven lamps may be left out of the circuit, and thus $\frac{1}{18}$ of 1000, $\frac{1}{9}$ of 500—55 milliamperes may be used.

And now we find that we have a more decided effect. By following each application with a galvanic battery by another with a faradic battery, we often secure admirable sedative results.

The X-rays, in the treatment of diseased organs, have proved efficacious in lupus, in securing epilation, in the solution of gall-stones, and other oily or fatty substances. Cancers have also been benefited by the X-ray, if we may judge by the results reported by foreign papers.

The X-ray in surgery: In Chicago, Harriet Heilmuth, a child five years old, and for two years blind and a paralytic, has had her sight and the use of her limbs restored through the agency of the X-rays. Two years ago, while playing, she fell, striking on her head. The fall left her blind and with her right side paralyzed. A few weeks ago the X-rays were applied, and revealed a tumor the size of an egg pressing on the brain. The skull was trephined directly over the cyst, as shown in the skiagraph, and the tumor was removed. The child was able to move her limbs on regaining consciousness.—*Christian Observer*, October 18, 1899.

More important to surgeons may be the galvano-cautery, with the many devices used for snares and blades, and curettes. In polypi of the nose the galvano-cautery has held a high place for many years.

In curetting indolent ulcers and tortuous suppurating sinuses, a small, dull curette can often be introduced after cocainizing the tract, and the current can be made to so quickly pass the platinum curette that a long operation may be avoided.

Catheterization of the fallopian tubes, in cases of pyosalpinx, has been followed by cures when the galvanic current was introduced at intervals sufficient to neutralize the effect of the pyogenic cocci.

The names of such men as Engelmann, Munde, Skene, Keith, Martin, Massey and others will always shine as masters of the new method of electrical treatment, but to Sprague belongs the credit of the successful treatment of pyosalpinx by electricity by catheterization.

Varicocele and hydrocele are maladies which may be relieved by electricity while hernia may be reduced under the faradic current. The vaso-motor nerves are influenced so as to slow the flow of blood to such an extent as to coagulate at the positive pole, while the negative pole may be capable of dissolving blood clots.

In this respect also the faradic current may induce contraction of the uterus in cases of post-partum hemorrhage and secure subinvolution in all relaxed conditions of the uterus.

According to Betton Massey, labor itself, at full term, may be hastened by the use of the faradic current which causes the pains of labor to be less, but which supplies the necessary contraction to the muscles independently. There may be some therapeutic value attached to this suggestion in cases of inertia of the womb in long labors.

